# Number of Medical Colleges in the United States

**Published:** 1858-09

**Authors:** 


### NUMBER OF MEDICAL COLLEGES IN THE UNITED STATES.

According to the *American Medical Gazette*, there are now forty-one medical colleges in our country. They are distributed as follows: In New York, six; Pennsylvania, four; Georgia, four; Ohio, three; Tennessee, three; Virginia, two; Kentucky, two; Missouri, two; Louisiana, two; Massachusetts, two; Vermont, three; Maine, one; New Hampshire, one; Connecticut, one; Iowa, one; Michigan, one; Illinois, one; Maryland, one; South Carolina, one.

